# Supplementary material for: What factors influence innovation efficiency in integrating digitalization and low carbonization within the construction industry? A configuration analysis based on fsQCA
Source: PLoS One. 2025 Mar 3;20(3):e0316249. doi: 10.1371/journal.pone.0316249 (PMC11875344; doi:10.1371/journal.pone.0316249)
Supplement: S1 Data — This is the Figures 2 and 3 title. (DOCX) [file pone.0316249.s001.docx]

Data of figure 2

| city | Technological innovation level of affiliation | Environmental regulation of affiliation |
| --- | --- | --- |
| Beijing | 1 | 1 |
| Jiangsu | 1 | 0.99 |
| Zhejiang | 0.97 | 0.98 |
| Hubei | 0.79 | 0.57 |
| Shanghai | 0.69 | 0.9 |
| Guangdong | 0.66 | 0.44 |
| Shandong | 0.57 | 0.46 |
| Anhui | 0.54 | 0.59 |
| Hebei | 0.51 | 0.71 |
| Shaanxi | 0.51 | 0.94 |

Data of figure 3

|  | ～Government Financial Support of affiliation | Environmental regulation of affiliation |
| --- | --- | --- |
| Fujian | 0.95 | 0.9 |
| Zhejiang | 0.72 | 0.98 |
| Hebei | 0.51 | 0.71 |
| Guangxi | 0.88 | 0.99 |
| Chongqing | 0.51 | 0.98 |
| Heilongjiang | 0.54 | 0.98 |
