# Supplementary material for: What factors influence innovation efficiency in integrating digitalization and low carbonization within the construction industry? A configuration analysis based on fsQCA
Source: PLoS One. 2025 Mar 3;20(3):e0316249. doi: 10.1371/journal.pone.0316249 (PMC11875344; doi:10.1371/journal.pone.0316249)
Supplement: S3 Data — This is the Table 7, title. (DOCX) [file pone.0316249.s003.docx]

Summary of data in the eastern region

| city | Technological Innovation Level（CX） | Technological Talent（RC） | Government Financial Support（CZ） | Government publicity and promotion（TG） | Environmental regulation（GZ） | Efficiency of integration of digitalization and low-carbonization innovation in the construction industry（RH） |
| --- | --- | --- | --- | --- | --- | --- |
| Beijing | 149.000 | 33.830 | 0.048 | 20.000 | 191.112 | -0.519 |
| Tianjin | 59.000 | 10.299 | 4.068 | 10.000 | 23.871 | -7.877 |
| Hebei | 61.000 | 12.561 | 0.028 | 14.000 | 45.423 | -3.467 |
| Liaoning | 107.001 | 11.651 | 0.023 | 12.000 | 22.997 | -11.057 |
| Shanghai | 82.000 | 23.551 | 0.445 | 9.000 | 47.961 | -2.844 |
| Jiangsu | 150.000 | 75.590 | 0.013 | 25.000 | 134.224 | -1.577 |
| Zhejiang | 156.000 | 57.528 | 0.017 | 13.001 | 67.225 | -1.945 |
| Fujian | 29.000 | 23.542 | 0.009 | 6.000 | 67.104 | -2.856 |
| Shandong | 150.000 | 44.764 | 0.020 | 17.000 | 44.295 | -4.045 |
| Guangdong | 126.000 | 88.525 | 0.011 | 35.000 | 46.838 | -4.155 |
| Hainan | 1.000 | 1.346 | 0.170 | 12.000 | 11.341 | -11.617 |

The eastern region variable calibrates the anchor point

| Category of variable | Primary Indicator | Secondary indicator | Calibration Anchor Points | | |
| --- | --- | --- | --- | --- | --- |
|  |  |  | Fully Affiliated Points | Intersection Points | Completely Unaffiliated Point |
| Antecedent Conditions | Technological conditions | Technological Innovation Level（CX） | 150.000 | 107.000 | 59.000 |
|  |  | Technological Talent（RC） | 57.528 | 23.552 | 11.651 |
|  | Organizational Conditions | Government Financial Support（CZ） | 0.170 | 0.024 | 0.013 |
|  |  | Government publicity and promotion（TG） | 20.000 | 13.000 | 10.000 |
|  | Environmental conditions | Environmental regulation（GZ） | 67.225 | 46.839 | 23.871 |
| Outcome variable | Efficiency of integration of digitalization and low-carbonization innovation in the construction industry（RH） | | -1.945 | -3.466 | -7.877 |

Summary of data in the central region

| city | Technological Innovation Level（CX） | Technological Talent（RC） | Government Financial Support（CZ） | Government publicity and promotion（TG） | Environmental regulation（GZ） | Efficiency of integration of digitalization and low-carbonization innovation in the construction industry（RH） |
| --- | --- | --- | --- | --- | --- | --- |
| Anhui | 78.000 | 23.529 | 0.029 | 24.000 | 43.566 | -3.734 |
| Jiangxi | 17.000 | 12.478 | 0.029 | 9.000 | 106.011 | -1.265 |
| Shanxi | 19.000 | 5.723 | 0.030 | 3.000 | 47.154 | -3.087 |
| Inner Mongolia | 15.000 | 2.643 | 0.502 | 48.000 | 4.009 | -57.673 |
| Henan | 51.000 | 22.243 | 0.023 | 68.000 | 36.116 | -4.823 |
| Hubei | 59.000 | 23.067 | 0.015 | 29.000 | 56.203 | -3.761 |
| Hunan | 42.000 | 20.933 | 0.009 | 22.000 | 34.640 | -6.052 |
| Jilin | 21.000 | 5.082 | 0.031 | 17.000 | 17.102 | -6.599 |
| Heilongjiang | 51.000 | 4.864 | 0.094 | 26.000 | 41.397 | -2.094 |
| Guangxi | 26.000 | 5.582 | 0.097 | 0.000 | 66.569 | -1.684 |

The central region variable calibrates the anchor point

| Category of variable | Primary Indicator | Secondary indicator | Calibration Anchor Points | | |
| --- | --- | --- | --- | --- | --- |
|  |  |  | Fully Affiliated Points | Intersection Points | Completely Unaffiliated Point |
| Antecedent Conditions | Technological conditions | Technological Innovation Level（CX） | 53.000 | 34.000 | 18.500 |
|  |  | Technological Talent（RC） | 22.449 | 9.101 | 5.027 |
|  | Organizational Conditions | Government Financial Support（CZ） | 0.095 | 0.030 | 0.021 |
|  |  | Government publicity and promotion（TG） | 33.750 | 23.000 | 7.500 |
|  | Environmental conditions | Environmental regulation（GZ） | 58.795 | 42.481 | 30.225 |
| Outcome variable | Efficiency of integration of digitalization and low-carbonization innovation in the construction industry（RH） | | -1.991 | -3.747 | -6.189 |

Summary of data in the western region

| city | Technological Innovation Level（CX） | Technological Talent（RC） | Government Financial Support（CZ） | Government publicity and promotion（TG） | Environmental regulation（GZ） | Efficiency of integration of digitalization and low-carbonization innovation in the construction industry（RH） |
| --- | --- | --- | --- | --- | --- | --- |
| Chongqing | 23.000 | 12.345 | 0.196 | 8.000 | 75.172 | -2.052 |
| Sichuan | 61.000 | 19.714 | 0.026 | 17.000 | 35.647 | -5.843 |
| Guizhou | 18.000 | 4.308 | 0.022 | 23.000 | 35.192 | -4.107 |
| Yunnan | 33.000 | 5.888 | 0.061 | 7.000 | 40.166 | -3.681 |
| Shaanxi | 45.000 | 12.528 | 0.015 | 45.000 | 61.951 | -2.583 |
| Gansu | 14.000 | 3.325 | 0.055 | 6.000 | 26.412 | -6.078 |
| Qinghai | 5.000 | 0.520 | 0.235 | 5.000 | 11.651 | -14.770 |
| Ningxia | 13.000 | 1.593 | 0.164 | 6.000 | 14.892 | -11.794 |
| Xinjiang | 11.000 | 1.924 | 0.052 | 17.000 | 19.290 | -11.417 |

The western region variable calibrates the anchor point

| Category of variable | Primary Indicator | Secondary indicator | Calibration Anchor Points | | |
| --- | --- | --- | --- | --- | --- |
|  |  |  | Fully Affiliated Points | Intersection Points | Completely Unaffiliated Point |
| Antecedent Conditions | Technological conditions | Technological Innovation Level（CX） | 36.000 | 18.000 | 12.500 |
|  |  | Technological Talent（RC） | 12.391 | 4.308 | 1.842 |
|  | Organizational Conditions | Government Financial Support（CZ） | 0.172 | 0.055 | 0.025 |
|  |  | Government publicity and promotion（TG） | 18.500 | 8.000 | 6.000 |
|  | Environmental conditions | Environmental regulation（GZ） | 45.612 | 35.192 | 18.190 |
| Outcome variable | Efficiency of integration of digitalization and low-carbonization innovation in the construction industry（RH） | | -3.406 | -5.843 | -11.511 |
