# Supplementary material for: What factors influence innovation efficiency in integrating digitalization and low carbonization within the construction industry? A configuration analysis based on fsQCA
Source: PLoS One. 2025 Mar 3;20(3):e0316249. doi: 10.1371/journal.pone.0316249 (PMC11875344; doi:10.1371/journal.pone.0316249)
Supplement: Attachment — Submitted filename: Response to Reviewers.docx [file pone.0316249.s005.docx]

Dear Experts and Teachers

Thank you for your decision and constructive feedback on our manuscript "What factors influence innovation efficiency in integrating digitalization and low carbonization within the construction industry? - A configuration analysis based on fsQCA" (ID:PONE-D-24-40808).This will certainly help us improve the quality of our manuscript. We have carefully considered the reviewers' suggestions and made revisions accordingly, as detailed below.

Note: In the response letter, all replies are displayed in black font, all changes and additions are highlighted in yellow, and deleted content is shown with red strikethrough. All reviewer comments are displayed in blue font. Additionally, all changes and additions in the revised manuscript are highlighted in yellow, and deleted content is shown with red strikethrough.

Wishing you a smooth workday and a fulfilling life!

Yours sincerely

Haifeng Xiong

**Journal Requirements:**

1.Please ensure that your manuscript meets PLOS ONE's style requirements, including those for file naming.

**Response:**Thank you sincerely for your advice. The article has been revised according to the style requirements of PLOS ONE. Please see the "Revised Manuscript with Track Changes" or "Manuscript" for the modified content.

2.Please provide an amended statement that declares *all* the funding or sources of support (whether external or internal to your organization) received during this study.Please also include the statement “There was no additional external funding received for this study.” in your updated Funding Statement. Please include your amended Funding Statement within your cover letter.

**Response:**Thank you sincerely for your advice. I have added the revised funding statement in the cover letter.

**The revised content is as follows:**

**Revised Statement of Funding:**This research was supported by "Basic Scientific Research Project of Liaoning Provincial Department of Education in 2022" (LJKMR20220709) and "General Project of School Social Science in 2023" (23-A045).There was no additional external funding received for this study.

3.Please confirm at this time whether or not your submission contains all raw data required to replicate the results of your study.Authors do not need to submit their entire data set if only a portion of the data was used in the reported study.If your submission does not contain these data, please either upload them as Supporting Information files or deposit them to a stable, public repository and provide us with the relevant URLs, DOIs, or accession numbers.

**Response:**Thank you sincerely for your advice.The data set for this article has been uploaded to the Dryad Digital Repository. DOI：<https://doi.org/10.5061/dryad.xsj3tx9r6>

4.When completing the data availability statement of the submission form, you indicated that you will make your data available on acceptance. We strongly recommend all authors decide on a data sharing plan before acceptance, as the process can be lengthy and hold up publication timelines. Please note that, though access restrictions are acceptable now, your entire data will need to be made freely accessible if your manuscript is accepted for publication. This policy applies to all data except where public deposition would breach compliance with the protocol approved by your research ethics board. If you are unable to adhere to our open data policy, please kindly revise your statement to explain your reasoning and we will seek the editor's input on an exemption. Please be assured that, once you have provided your new statement, the assessment of your exemption will not hold up the peer review process.

**Response:**Thank you sincerely for your advice. The data set of this article has been uploaded to Dryad Digital Repository for data sharing.

5.PLOS requires an ORCID iD for the corresponding author in Editorial Manager on papers submitted after December 6th, 2016. Please ensure that you have an ORCID iD and that it is validated in Editorial Manager. To do this, go to ‘Update my Information’ (in the upper left-hand corner of the main menu), and click on the Fetch/Validate link next to the ORCID field. This will take you to the ORCID site and allow you to create a new iD or authenticate a pre-existing iD in Editorial Manager.

**Response:**Thank you sincerely for your advice, ORCID iD of the corresponding author is validated in Editorial Manager

**Reviewer #1:**

(1)The introduction does a good job of framing the study within the dual contexts of digitalization and the “double carbon” goal. However, the discussion on how the construction industry’s carbon emissions align with these global and national goals could be expanded. Adding specific challenges faced by the industry in integrating digitalization and low carbonization would strengthen the relevance of your research.

**Response:**Thank you sincerely for your thorough review of our paper. The introduction has now been updated to include a discussion on the consistency between carbon emissions in the construction industry and global and national targets. Additionally, three specific challenges faced by the construction industry in the integration of digitalization and low-carbon development have been added. Furthermore, the data in the introduction has been updated to reference the latest authoritative figures. Due to the extensive nature of the modifications, please refer to the "Revised Manuscript with Track Changes" for specific changes.

(2)The statement of the problem should explicitly highlight what existing literature lacks concerning the integration of digital and low-carbon innovations. Clearly stating the three key questions in bullet points would also improve readability.

**Response:**Thank you sincerely for your thorough review of our paper.The shortcomings of existing literature on the integration of digital and low-carbon innovation have now been emphasized at the beginning of the third paragraph of the introduction, and the descriptions of the three key issues at the end have been further refined and supplemented.

**The revised content is as follows:**

……This article aims to address the following questions: (1) Whether the five antecedent conditions in the TOE framework—technology innovation level, technical talent, government financial support, government promotion, and environmental regulation—exist as necessary conditions that can lead to high efficiency in the integration of digitalization and low carbonization in the construction industry?(2) Whether the five antecedent conditions, namely, technological innovation level, technological talents, governmental financial support, governmental publicity and promotion, and environmental regulation, can be configured in several grouping paths that adequately result in high construction industry digitalization and low carbonization integration innovation efficiency?(3) Can the five antecedent conditions of technological innovation level, technological talents, governmental financial support, governmental publicity and promotion, and environmental regulation form several grouping paths that could be used to generate Non-high innovation efficiency in the integration of digitalization and low carbonization in the construction industry?

(3)The current review mixes several themes, such as digitalization, low carbonization, and BIM technology. I recommend separating these into distinct subsections (e.g., digitalization in construction, low carbonization initiatives, and synergies between them). This would make the literature review easier to follow and emphasize the progression of research

**Response:**Thank you sincerely for your thorough review of our paper.After considering the experts' suggestions, the "Literature Review" has been divided from the original single chapter into four smaller sections based on different content, and multiple references have been added according to the divided sections.Due to the extensive nature of the modifications, please refer to the "Revised Manuscript with Track Changes" for specific changes.

(4)While many studies are mentioned, the review lacks critical engagement with these sources. You should critically assess how past research either supports or contradicts your findings.

**Response:**Thank you sincerely for your thorough review of our paper.The article has critically assessed the literature review from three aspects. Due to the extensive nature of the revisions, the specific changes can be found in the "Revised Manuscript with Track Changes" under the section for Literature Review.

(5)It would be helpful to provide a more detailed explanation of why fsQCA is better suited to this study than other quantitative methods. How does it handle the complexity of multiple interacting factors better than regression-based models?

**Response:**Thank you sincerely for your thorough review of our paper.The reasons why fsQCA is more suitable for this research than other quantitative methods have now been added to the "Qualitative Comparative Analysis Methods" chapter.

**The revised content is as follows:**

……it is generally believed that the result is produced by the concurrent influence of multiple factors[12].

This article abandons the traditional symmetry research methods and chooses the fuzzy set qualitative comparative analysis (fsQCA) method from QCA for the following reasons: traditional regression models typically assume that the relationships between variables are symmetric and linear. However, in the process of integrating digitalization and low carbonization in the construction industry, the influence of multiple antecedent conditions is not a linear relationship. fsQCA can handle these nonlinear and complex interactions. By analyzing the changes in outcomes under different combinations of interacting factors, it reveals the nonlinear relationships between them, thus providing a more accurate and comprehensive explanation. In addition, fsQCA employs a multi-case study approach, which can fully take into account the heterogeneity and complexity of cases across different provinces. It is based on the ideas of set theory and configurational thinking, examining the relationship between antecedent conditions and outcomes from the perspective of sets. By establishing a multivariate analysis of causal relationships, fsQCA can identify the configurations of antecedent conditions that influence the integration of digitalization and low carbonization innovation in the construction industry, and explore the equivalence between different antecedent configurations. fsQCA allows variables to take multiple continuous values between 0 and 1, which means that the variables are no longer "black or white," but rather have different degrees. This application of fuzzy sets enables fsQCA to more accurately reflect the ambiguity and uncertainty in the construction industry's process of digitalization and low carbonization.

(6)it would be helpful to deepen the theoretical implications of your findings. You could also discuss how your findings challenge or extend existing theories in digital and low-carbon innovation.

**Response:**Thank you sincerely for your thorough review of our paper.The theoretical significance of this article has now been added to the "Conclusions and limitations" section.

**The revised content is as follows:**

**Theoretical significance**

firstly, when analysing the complex issue of digital transformation and low carbonization development of the construction industry, this paper employs the TOE theoretical framework in a clever and integrated manner, constructing a comprehensive and in-depth research framework with the aim of systematically exploring the key factors affecting the efficiency of the integration of digitalization and low carbonization innovation in the construction industry. The TOE theoretical framework originally emphasises the impact of technological factors, internal organizational factors and external environmental factors on corporate strategy and performance. The TOE theoretical framework originally emphasises the influence of technological factors, internal organisational factors, and external environmental factors on corporate strategy and performance. This paper builds on this by incorporating the construction industry's unique industry characteristics, policy orientation, and market demand, thus forming a set of analyses that is both universal and industry-specific. This research framework offers academics and practitioners a systematic and comprehensive understanding of the multi-dimensional influencing factors of the innovation efficiency of the integration of digitalization and low carbonization in the construction industry. Furthermore, it provides novel research perspectives and theoretical support for subsequent scholars' research in this field, thereby facilitating the deepening and expansion of academic research.

Secondly, a significant methodological contribution of this study is the introduction of the group perspective, which represents a noteworthy theoretical advancement in the study of the factors influencing the efficiency of the integration of digitalization and low carbonization in the construction industry. This perspective allows the study to analyse not only the direct effect of a single factor on the convergence innovation efficiency, but also, more importantly, to reveal the multiple path effects of multiple factors in different configurations and combinations. This provides a powerful analytical tool to address the complexity and uncertainty inherent in the process of convergence of digitisation and low carbonization in the construction industry. Furthermore, this research methodology offers practical guidance on how the construction industry can enhance the innovation efficiency of digitalization and low carbonization integration. It encourages enterprises and organisations in the industry to consider not only the introduction of a single technology or the implementation of a policy, but also the synergy between factors as a whole and the combination of paths that is most suitable for their own development when promoting change. This provides new perspectives and a solid foundation for the realisation of a green, smart and sustainable construction industry. The objective is to provide new perspectives and a solid theoretical basis for the realisation of green, intelligent and sustainable development of the construction industry.

(7)A thorough proofreading for grammar and consistency will improve the overall quality of the manuscript.

**Response:**Thank you sincerely for your thorough review of our paper.The grammar and consistency of the entire text have now been checked and improved.Due to the extensive nature of the modifications, please refer to the "Revised Manuscript with Track Changes" for specific changes.

**Reviewer #2:**

(1)This study uses cross-sectional data from 30 provinces, which is a relatively small sample size. How do the authors ensure the representativeness of the selected sample? The authors should discuss potential biases arising from sample selection.

**Response:**Thank you sincerely for your thorough review of our paper.The manuscript has supplemented the explanation of sample diversity from the perspectives of geographical distribution and economic and social development. It illustrates the representativeness of the 30 provincial cases from three aspects: regional distribution, economic development status, and data acquisition channels.

**The revised content is as follows:**

**Data collection and processing**

This article selects sample data from 31 provinces (autonomous regions and municipalities) in China. Considering the completeness of the data, the Tibet Autonomous Region, which lacks environmental regulation data, was excluded. The remaining 30 provinces cover the geographical locations of China's eastern, central, and western regions, including economically developed provinces such as Beijing and Shanghai, as well as underdeveloped provinces like Qinghai and Gansu, representing a variety of sample types. The sample data mainly comes from the following aspects.The data on the antecedent conditions for technological innovation were sourced from the China Patent Star Search System. Information regarding technological talent was extracted from the 2022 China Science and Technology Statistical Yearbook. The official websites of the provincial governments, as well as the 2022 China Statistical Yearbook, were consulted to gather data on the government’s financial support and promotional efforts for the innovative concept of digitalization and low carbonization in the construction industry. The data on environmental regulation were sourced from the 2022 China Energy Statistics Yearbook and the 2022 China Statistical Yearbook. The innovation efficiency of digitalization and low carbonization integration in the construction industry was obtained from the 2022 China Energy Statistics Yearbook and the 2022 China Statistical Yearbook.

(2) Although the fsQCA method can identify combinations of conditions, it is difficult to establish strict causal relationships. However, judging from the paper's title, the authors clearly intend to discuss the factors influencing the innovation efficiency of digitalization and low carbonization integration in the construction industry.

**Response:**First of all, I sincerely thank you for your detailed review of my paper and for your valuable comments on the application of the fsQCA method. You pointed out the advantages of the fsQCA method in identifying combinations of conditions, but also raised its limitations in establishing strict causal relationships, which is indeed a topic worth exploring in depth.

In my research, I chose the fsQCA method primarily because of its ability to handle complex causal relationships and combinations of conditions. Through fsQCA, I was able to identify the interactions among different conditions such as technology, organization, and environment, as well as their joint impact on the efficiency of digitalization and low-carbon integration innovation in the construction industry. This method provided me with a new perspective to examine this complex issue and helped me discover some interesting and meaningful results.

Regarding the difficulties you mentioned in establishing strict causal relationships, I fully understand. Indeed, the fsQCA method focuses more on the analysis of the associations between combinations of conditions and outcomes rather than strict causal inference. However, I believe this does not diminish the application value of the fsQCA method in this study. My main goal in this research is to explore the key factors influencing the efficiency of digitalization and low-carbon integration innovation in the construction industry and to understand the interactions among these factors. The fsQCA method serves as a powerful analytical tool for this purpose.

Once again, thank you for your review and valuable comments. Your guidance is a precious asset to me. I will carefully consider your suggestions and continue to explore and refine my research methods in future studies.

(3)The use of static cross-sectional data overlooks the dynamic characteristics of innovation efficiency over time. Why didn't the paper consider panel data over multiple years?

**Response:**Thank you very much for your detailed review of my paper and for raising important points regarding data selection. You pointed out that static cross-sectional data may overlook the dynamic characteristics of innovation efficiency as it changes over time, which is a profound and worthy issue to explore.

In this study, I chose to use static cross-sectional data primarily based on a comprehensive consideration of the current research objectives and data availability. This research aims to explore the impact of factors such as technology, organization, and environment on the integration of digitalization and low carbonization innovation efficiency in the construction industry at a specific point in time. By using cross-sectional data, we can capture the interactions of these factors at a given moment, providing valuable insights into the current state of innovation efficiency.

Regarding the use of panel data, I fully understand that it can capture the trends of variable changes over time, thereby revealing the dynamic characteristics of innovation efficiency more accurately. However, in this study, due to limitations in data acquisition, particularly the scarcity of detailed long-term data on digitalization and low carbonization in the construction industry, I opted for the most recent and comprehensive cross-sectional data available for the research. Of course, I recognize that panel data will have significant value in future research, and I plan to continue exploring this direction in my future work to gain a deeper understanding of the dynamic characteristics of innovation efficiency.

(4)The paper lacks heterogeneity analysis of differences between provinces.

**Response:**Thank you sincerely for your thorough review of our paper.This article has now been supplemented with a heterogeneity analysis of inter-provincial differences, analyzing the heterogeneity of provinces in three regions: the eastern region, the central region, and the western region.Due to the extensive nature of the modifications, please refer to the "Revised Manuscript with Track Changes" for specific changes.

(5)The policy recommendations based on the research results are relatively general and lack specific, actionable measures.

**Response:**Thank you sincerely for your thorough review of our paper.The article has now supplemented specific and actionable measures based on the research findings for policy recommendations.Due to the extensive nature of the modifications, please refer to the "Revised Manuscript with Track Changes" for specific changes.

(6)The discussion on the limitations of the fsQCA method itself is insufficient, such as the sensitivity of the variable calibration process.

**Response:**Thank you sincerely for your thorough review of our paper.The article has added a discussion on the limitations of the fsQCA method in the final chapter titled "Shortcomings and Prospects."

**The revised content is as follows:**

**Shortcomings and prospects**

(1) Due to the limited research and insufficient statistical data on the integration and innovation of digitalization and low carbonization in the construction industry, this article only uses static data from 2021. Future researchers can collect dynamic data from the past 5 to 10 years and analyze it using the QCA method. (2) In this study, the fsQCA research method was employed. The calibration process of fsQCA is highly sensitive, and different calibration methods and parameter settings may yield disparate analysis results, thereby increasing the complexity and unpredictability of the findings. In contrast, NCA can provide a more stable datum point for the analysis by identifying the necessary conditions. It is recommended that in the future, scholars employ the fsQCA method in conjunction with the NCA method to mitigate the uncertainty inherent in fsQCA analyses by leveraging the stability of NCA to enhance the precision and dependability of the outcomes. (3) This article summarizes the experiences and research of predecessors and selects five important aspects as antecedent conditions. In the future, research can be conducted on the integration of digitalization and low carbonization innovation efficiency in the construction industry by adding or changing these antecedent conditions.

(7)There are grammatical issues, for example, in the paper title "What influences the innovation efficiency of digitalization and low carbonization integration in the construction industry? -Configuration analysis based on fsQCA", an article "a" should be added before "Configuration analysis".

**Response:**Thank you sincerely for your thorough review of our paper.The grammar and consistency of the entire text have now been checked and improved.Due to the extensive nature of the modifications, please refer to the "Revised Manuscript with Track Changes" for specific changes.

(8)Some figures and tables in the paper are not standardized. For instance, Figure 2 does not explain what the x and y axes represent.

**Response:**Thank you sincerely for your thorough review of our paper.The tables and figures in the article have been standardized, and the meanings of the horizontal and vertical axes have been added to Figures 2 and 3.Please refer to the "Revised Manuscript with Track Changes" for specific changes.
